# Comparative genome analysis of the genus *Marivirga* and proposal of two novel marine species: *Marivirga arenosa* sp. nov., and *Marivirga salinae* sp. nov.

**DOI:** 10.1186/s12866-024-03393-3

**Published:** 2024-07-05

**Authors:** Neak Muhammad, Forbes Avila, Song-Gun Kim

**Affiliations:** 1 Biological Resource Center/Korean Collection for Type Cultures (KCTC), Korea ResearchInstitute of Bioscience and Biotechnology, Jeongeup, Jeonbuk 56212 the Republic of Korea; 2 Department of Biotechnology, KRIBB School, University of Science and Technology (UST), Daejeon, 34113 the Republic of Korea

**Keywords:** *Bacteroidota*, KEGG, CAZymes, Polysaccharide, DNRA, Heavy metals

## Abstract

**Background:**

The phylum *Bacteroidota* represents a significant proportion of heterotrophic bacteria found in marine ecosystems. Members of the phylum *Bacteroidota* are actively involved in the degradation of biopolymers such as polysaccharides and proteins. *Bacteroidota* genomes exhibit a significant enrichment of various enzymes, including carbohydrate-active enzymes (CAZymes), carboxypeptidases, esterases, isomerases, peptidases, phosphatases, and sulfatases. The genus *Marivirga*, a member of the family *Marivirgaceae* within the phylum *Bacteroidota*, comprises six documented species. During a microbial diversity study, three novel *Marivirga* strains (BKB1-2^ T^, ABR2-2, and BDSF4-3^ T^) were isolated from the West Sea, Republic of Korea.

**Results:**

To explore the taxonomic status and genomic characteristics of the novel isolates, we employed a polyphasic taxonomic approach, which included phylogenetic, chemotaxonomic and comprehensive genome analysis. The three isolates were Gram-stain-negative, aerobic, rod-shaped, moderately halophilic, and had a gliding motility. The average nucleotide identity (ANI) and digital DNA-DNA hybridization (dDDH) values among the two isolates, BKB1-2^ T^ and BDSF4-3^ T^, and the six reference strains were 70.5–76.5% for ANI and 18.1–25.7% for dDDH. Interestingly, the Kyoto Encyclopedia of Genes and Genomes (KEGG) analysis showed that the strains harbor genes for a comprehensive pathway for dissimilatory nitrate reduction to ammonium (DNRA), as well as other nitrogen pathways for the reduction of nitrite, nitric oxide, and nitrous oxide. Additionally, the antiSMASH analysis indicated that the strains contained three to eight biosynthetic gene clusters (BGCs) associated with the synthesis of secondary metabolites. Furthermore, the strains carried a high number of CAZyme ranging from 53 to 152, which was also demonstrated by an in vitro analysis of degradation of the polysaccharide cellulose, chitin, laminarin, starch, and xylan. Additionally, all the strains carried genes for the metabolism of heavy metals, and exhibited tolerance to heavy metals, with minimum inhibitory concentrations (MICs) in millimoles (mM) in ranges of Co^2+^ (3–6), Cu^2+^ (0.2–0.4), Ni^2+^ (3–5), Zn^2+^ (2–4), Mn^2+^ (20–50), and Hg^2+^ (0.3).

**Conclusions:**

Based on polyphasic taxonomic approach, the three isolated strains represent two novel species names *Marivirga arenosa* sp. nov. (BKB1-2^ T^ = KCTC 82989^ T^ = InaCC B1618^T^), and *Marivirga salinae* sp. nov. (BDSF4-3^ T^ = KCTC 82973^ T^ = InaCC B1619^T^).

**Supplementary Information:**

The online version contains supplementary material available at 10.1186/s12866-024-03393-3.

## Background

The phylum *Bacteroidota* constitutes a significant component of marine heterotrophic bacteria. After *Pseudomonatoda* and *Cyanobacteria*, members of the phylum *Bacteroidota* rank as the ocean's most abundant bacterial group [1]. *Bacteroidota* strains have been isolated from a wide range of marine environments, including tidal flats [2], coastal sediments [3], deep sea [4], and hydrothermal vents [5]. By analyzing metagenomic data obtained from global ocean sampling and conducting a CARD-FISH study across the North Atlantic Ocean it was revealed that, subsequent to *Cyanobacteria* and *Pelagibacter*, *Bacteroidota* are the prevailing bacteria in marine environments [6].

Members of the phylum *Bacteroidota* participate in the breakdown of biopolymers, including polysaccharides and proteins, in marine ecosystems [1]. *Bacteroidota* are abundant in algal and phytoplankton blooms, displaying a preference for the consumption of complex polysaccharides [7]. Deep-sea sediments, where marine plants and animals remains accumulate, also contain complex polysaccharides and proteins, along with a notable presence of *Bacteroidota* species [4]. Complex polysaccharides found in marine environments comprise a variety of compound including agar, alginate, chitin, carrageenan, cellulose, fucoidans, laminarin, pectin, porphyrin, and xylan [8]. The members of the phylum *Bacteroidota* efficiently degrade complex polysaccharides, thus contributing to global recycling of carbon [4].

A comparative genome analysis showed that the genomes of *Bacteroidota* are highly enriched for carbohydrate-active enzymes (CAZymes), suggesting their role in degrading complex polysaccharides [9, 10]. Second to CAZymes, the *Bacteroidota* genome harbors a significant number of enzymes, including carboxypeptidases, esterases, isomerases, peptidases, sulfatases, and phosphatases that are involved in the mineralization of high molecular weight (HMW) organic matter [11]. In addition, genome mining data revealed that strains within the phylum *Bacteroidota* are abundant in genes of secondary metabolite synthesis that have diverse pharmacological activities [12].

The phylum *Bacteroidota* consists of six classes, each with corresponding six orders. Among them, the order *Cytophagales* of the class *Cytophagia* comprise a total of 21 families (https://lpsn.dsmz.de/order/cytophagales). The family *Marivirgaceae* contains only one validly published genus, *Marivirga.* A total of six *Marivirga* species have been documented, namely*, M. sericea*, *M. tractuosa* [13, 14], *M. lumbricoides* [15], *M. atlantica*, *M. harenae* [16, 17], and *M. aurantiaca* [18]. The isolation sources include marine aquariums, sand, sea water, and sediments. They are Gram-negative rod, non-spore forming, and aerobic. The respiratory quinone is menaquinone 7.

In this study, we isolated three novel gliding bacterial strains (BKB1-2^ T^, ABR2-2, and BDSF4-3^ T^) from marine samples collected in the West Sea, Korea. Based on a polyphasic taxonomic approach employing phylogenetic and chemotaxonomic analyses and a comprehensive genome analysis, two new species in the genus *Marivirga* were proposed*.* The genomes of isolates and six existing strains of the genus *Marivirga* were comparatively analyzed. The analysis showed that the strains carried a high number of CAZymes and degraded various types of complex polysaccharides, including chitin, cellulose, laminarin, starch, and xylan, contributing to the nutrient cycle. Additionally, the strains harbor genes responsible for synthesizing various amino acids, vitamins, and secondary metabolites, managing heavy metal metabolism, and carrying various pathways crucial for the nitrogen cycle in coastal ecosystems. Overall, the genomics analysis emphasized the strains' contributions at both environmental and industrial levels.

## Methodology

### Sampling sites

As part of a microbial diversity study, multiple marine samples were collected at various locations in the West Sea, Republic of Korea, in June 2021. Three types of samples were primarily collected: sea sand, green alga of the genus *Ulva*, and microbial mats, each with distinct ecological importance. A sample from sea sand was collected from an estuary located on Docho Island (34° 41′ 21.40″ N, 125° 55′ 08.46″ E). The sea sand in the estuary is influenced by both marine and freshwater systems, experiencing varying salinity levels and fostering a unique microbial community. A sample from green alga belonging to the genus *Ulva* was collected on Aphae Island (34° 50′ 43.42" N, 126° 13′ 32.19" E). The algae provide a habitat for diverse microbial communities, particularly for polysaccharide degraders, contributing to nutrient cycling. A sample from microbial mats was collected from a saltern on Bigeum Island (34° 45′ 49.35" N, 125° 58′ 42.48" E). The microbial mats from the saltern, a highly saline environment, form complex communities of cyanobacteria, algae, and various bacteria and archaea. These varied environments highlight the rich microbial diversity and ecological significance of the samples collected. All the samples were promptly transferred and processed at the laboratory.

### Sample processing

To isolate more novel strains, we aimed to replicate natural conditions: seawater from the sampling area was collected and used in the preparation of media. A small part of each sample was placed at the center of a low-nutrient solid medium composed of 60% seawater, 1.5% agar, and 50 mg/L filtrate-sterilized cycloheximide. The plates were kept in a 20 °C incubator for seven days and observed using a stereoscopic microscope (ZEISS Stemi 508). The gliding colonies were transferred onto rich media of marine agar (MA, BD) and modified VY/2 agar media [MVY; 60% (v/v) seawater, 5 g/L baker’s yeast (Sigma), and 25 mg/L vitamin B_12_] [19]. The colonies were further purified and preserved at -80 °C in 20% glycerol. Finally, the isolated strains BKB1-2^ T^, ABR2-2, and BDSF4-3^ T^ were deposited at the Korean Collection for Type Cultures (KCTC) and in the Indonesian Culture Collection (InaCC).

### 16S rRNA gene sequencing

The 16S rRNA gene was amplified with the universal bacterial primer pairs, 27F and 1492R. The gene was sequenced using the Sanger sequencing method with universal bacterial primers and two additional primers, 518F and 518R [20]. The near-complete 16S rRNA gene sequences were assembled by Vector NTI software. The sequences were then searched on the EzBioCloud database to find similar sequences [21]. The sequences obtained from the EzBioCloud database were aligned, trimmed, and then edited using BioEdit software (version 7.2.5). The number of nucleotides used for the construction of the phylogenetic tree was 1334 bp. Finally, three phylogenetic trees were made using molecular evolutionary genetics analysis (MEGA X) software [22], employing the neighbor-joining (NJ) [23], the maximum-likelihood (ML) [24], and the maximum parsimony (MP) methods [25]. The robustness of the phylogenetic trees was assessed with the 1,000 bootstrap iterations method. *Flexibacter flexilis* NBRC 15060^ T^ (AB680763) was used as an outgroup.

### Physical, biochemical and chemotaxonomic analysis

To determine characteristics, three isolated strains were cultivated on MA. A BBL™ Gram staining kit (BD, USA) was used to perform Gram staining. The morphology and size of bacterial cells were determined using a scanning electron microscope (SEM) (Regulus 8100, Hitachi) [26]. All isolates were checked for gliding motility through the hanging-drop technique and using soft agar media [27]. The optimum temperature and salt concentration for growth were assessed by using MA as the growth medium [2], while the optimum pH was examined using pH adjusted marine broth (MB, BD) as the testing medium [28]. For the determination of growth under anaerobic conditions, all isolates were inoculated within an anaerobic chamber (Coy Labs, USA) and the cells were cultivated on MA in an anaerobic jar at 30 °C (BD GasPak Systems).

For biochemical characterization, the catalase and oxidase production were examined using 3% (v/v) H_2_O_2_ and 1% (w/v) tetramethyl- *p*-phenylenediamine dihydrochloride reagents, respectively [29]. The hydrolysis of Tweens 20, 40, 80, and casein were determined as described in the Cowan and Steel protocol [30]. The activities of the DNase enzyme were checked using DNase agar (BD). The presence of flexirubin pigments was assessed by using a 20% (w/v) KOH solution [31]. For the other biochemical characterizations, API 20E, API ZYM, API 50CH strips (bioMérieux), and GEN III MicroPlates (Biolog) were used [32]. To investigate dissimilatory nitrate/nitrite reduction to ammonium (DNRA) activities, MB was supplemented with 1.0 g/L (w/v) NaNO_3_. Three isolated strains, along with the reference strains in the genus *Marivirga*, were inoculated into media. After three days of incubation, MQuant colorimetric test strips (Merck, Darmstadt, Germany) were employed to quantify the ammonium content in the media [33]. Second to DNRA, the nitrite reduction capacity was assessed by utilizing MB supplemented with 5 mM NaNO_2._ The strains were inoculated into media and subsequently checked for the reduction of nitrite. Nitrite concentrations were measured using Griess reagent, which consisted of sulfanilamide and N-(1-naphthyl) ethylenediamine [34]. The assay was performed at three time points during the incubation period: at the onset (day 0), after three days, and after five days, with the aim of assessing changes in nitrite concentration within the media [18]. For the chemotaxonomic analysis, the fatty acid profiles of three isolated strains and reference strains were determined by cultivating cells on MA for two days The cellular fatty acid methyl esters were extracted following the MIDI protocol (Sherlock Microbial Identification System version 6.0) [35]. Subsequently, the extracted components were injected into a gas chromatography system (GC system 8890, Agilent) and then the components were identified based on the TSBA version 6.0 database. The quinones and polar lipid profiles were assessed using the Komagata and Suzuki protocol [36]. For the quinone determination, the compounds were extracted from freeze-dried cells using chloroform–methanol (2:1, v/v). The crude components were separated by thin layer chromatography (TLC) and then identified by an HPLC system. The compound was identified by comparing the retention time of the standard compound extracted from reference strains. For the polar lipids, the compounds were separated by a two-dimensional TLC plate (silica gel 60 F254, 10 × 10 cm, Merck) and then sprayed with different reagents: ninhydrin, molybdenum blue, α-naphthol, and phosphomolybdic acid for the detection of amino lipids, phospholipids, glycolipids, and total lipids, respectively [36].

### Genome sequencing and phylogeny

The genome sequences of three isolated strains, BKB1-2^ T^, ABR2-2, BDSF4-3^ T^, and one of the reference strains *M. harenae* KCTC 92433^ T^ were determined by Oxford Nanopore Technologies (ONT, United Kingdom). In the sequencing process, a ligation sequencing kit (SQK-LSK112), a barcoding kit (SQK-NBD112.24), an R10.4 FLO-MIN112 flow cell, and a MinION device were utilized. Basecalling was conducted using MinKNOW software version 22.10.7 and Guppy software version 6.3.8 [37]. The genome was then assembled with Flye version 2.9.1 [38]. The genome's contamination and completeness were evaluated using CheckM version 1.2.2 (github.com/Ecogenomics/CheckM) and Busco version 5.4.4 (busco.ezlab.org/) [39, 40].

The ANI and dDDH were determined using the ANI calculator and DSMZ’s genome to genome distance calculator version 3.0 [21, 41]. A phylogenomic tree was constructed using the up-to-date bacterial core gene (UBCG) pipeline, incorporating the 92 prokaryotic core genes [42]. *Flexibacter flexilis* DSM 6793^ T^ (GCF900112255) was used as an outgroup.

### Functional analysis of genome

The genomes of the three strains, BKB1-2^ T^, ABR2-2, and BDSF4-3^ T^, along with the reference strain *M. harenae* KCTC 92433^ T^, were deposited at NCBI and annotated utilizing NCBI’s prokaryotic genome annotation pipeline (PGAP) [43]. The metabolic pathways were identified using the KEGG and RAST databases. First, KEGG pathways were detected using the BlastKOALA (kegg.jp/blastkoala/) server. The results from the BlastKOALA server were then processed using a KEGG-decoder [44]. Furthermore, a heatmap was generated from the KEGG pathways employing GraphPad Prism version 8.0.2. The RAST server was used to further determine the metabolic diversity and functional capabilities of all species in the genus *Marivirga* [45]. The antiSMASH database was employed to detect the secondary metabolite genes called biosynthetic gene clusters (BGCs) [46]. The genomes were further analyzed for carbohydrate active enzymes (CAZymes) through the dbCAN2 database [47].

### Polysaccharide degradation testing

The degradation of complex polysaccharides was tested in basal agar media [60% (v/v) seawater, 0.1% sodium acetate, 0.01% (w/v) peptone, 0.06% (w/v) HEPES (pH xx), 0.01% (w/v) K_2_HPO_4_, 0.02% (w/v) NH_4_NO_3_, 1 ml trace elements, and multivitamins (https://www.dsmz.de/microorganisms/medium/pdf/DSMZ_Medium1579.pdf), containing 1% (w/v)) of polysaccharides, including alginate, cellulose, chitin, κ-carrageenan, λ-carrageenan, ι-carrageenan, laminarin, starch, and xylan [48, 49]. All three newly isolated strains were inoculated on the test media and incubated for one week at 30 °C. The breakdown of complex polysaccharides was identified by the formation of a clear zone surrounding the colonies.

To confirm the degradation of complex polysaccharides, we conducted further analyses in basal broth, mirroring the composition of the basal agar media. However, the media were modified to include 0.2% (w/v) of each test polysaccharide, encompassing alginate, cellulose, chitin, κ-carrageenan, λ-carrageenan, ι-carrageenan, laminarin, starch, and xylan [48, 49]. The degradation of polysaccharide in the broth media was confirmed by using 3, 5 dinitrosalicylic acid (DNS) reagent, which detects the reducing sugar released from the breakdown of polysaccharide [50]. The DNS reagent is added to the supernatant, and upon heating, it reacts with reducing sugars, causing a color change from yellow to orange. The DNS assay was performed at three time points of incubation: at the start (day 0), after two days, and then four days. During each of these time points, the color changes were subsequently measured at 570 nm using a microplate reader (Synergy H1, BioTek) [50].

### Determination of tolerance to heavy metals

Minimum inhibitory concentration (MIC) is the lowest concentration of an inhibitory substance at which bacterial growth is inhibited. Tolerance to heavy metals was measured by determining the lowest concentration of each metal that prevents the growth of the isolated strains [51]. All the strains were tested for tolerance of heavy metals including Cu^2+^, Co^2+^, Ni^2+^, Zn^2+^, Mn^2+^, and Hg^2+^. The CuSO_4_.5H_2_O, CoCl_2_.6H_2_O, NiCl_2_.6H_2_O, ZnCl_2_, MnCl_2._4H_2_O, and HgCl_2_ were used as sources of Cu^2+^, Co^2+^, Ni^2+^, Zn^2+^, Mn^2+^, and Hg^2^. For the determination of MIC, the MA medium was supplemented with mM concentrations of Cu^2+^ (0.1–1), Co^2+^ (1–7), Ni^2+^ (1–5), Zn^2+^ (1–5), Mn^2+^ (10–50), and Hg^2+^ (0.05–0.5) [52, 53]. All three isolates were inoculated in duplicate and then incubated for one week at 30 °C.

## Results and discussion

### Isolation of novel strains

**Fig. 1:**
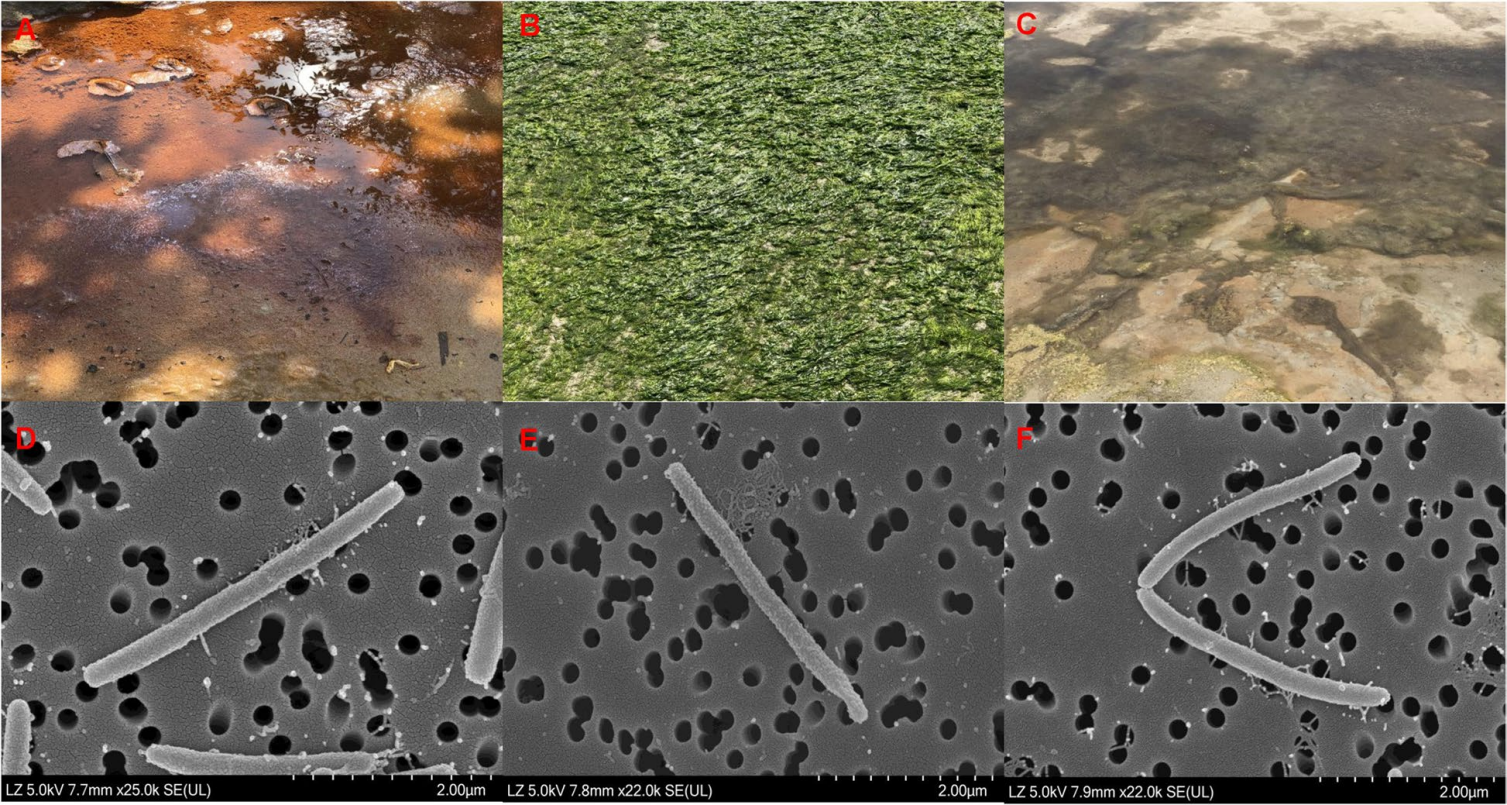
Sources of isolation (**A**, **B**, **C**), and SEM images (**D**, **E**, **F**) of three isolated strains. Strain: BKB1-2^ T^ (**A**, **D**); ABR2-2 (**B**, **E**); BDSF4-3.^T^ (**C**, **F**). Scale bars: 2 μm (**D**, **E**, **F**)

Many novel strains have been isolated from multiple marine samples. Some of the isolates have been characterized as belonging to genera *Reichenbachiella* [49], *Chondrinema* [54], *Fulvivirga* [19] *Flavobacterium* [28], and *Vibrio* [32]. In this study three more strains, namely BKB1-2^ T^, ABR2-2, and BDSF4-3^ T^, isolated from sea sand, green algae of the genus *Ulva*, and microbial mat, respectively, were characterized (Figs. 1A, B, C). All three strains grew optimally on MA and produced orange, smooth, and circular colonies. The SEM images showed the strains are long rods with a length of 3.0–3.7 μm and a diameter range from 0.2–0.3 μm (Figs. 1D, E, F).

### Phylogenetic analysis based on 16S rRNA sequence

A phylogenetic analysis using 16S rRNA gene sequence confirmed that the strains BKB1-2^ T^, ABR2-2, and BDSF4-3^ T^ were associated with the genus *Marivirga* within the phylum *Bacteroidota*, with the closest strains of BKB1-2^ T^ and ABR2-2 being *M. tractuosa* DSM 4126^ T^ with similarity values of 97.78% and 97.92%, while the closest strain of BDSF4-3^ T^ was *M. sericea* DSM 4125^ T^ with a similarity value of 98.68%. The 16S rRNA gene similarity value between BKB1-2^ T^ and ABR2-2 was 99.8%, indicating that strains BKB1-2^ T^ and ABR2-2 belong to the same species (Table S1). The similarity values of BKB1-2^ T^ and BDSF4-3^ T^ against the reference strains in the genus *Marivirga* fell below the threshold value of species description of 98.7% [55]. Furthermore, we compared the sequence of the 16S rRNA gene obtained from Sanger sequencing with the sequences obtained from whole genome sequencing. The 16S rRNA sequences from both sources were aligned using BioEdit to assess similarity, which were ranged from 99.95–99.98%. For further analysis, the 16S rRNA sequence from Sanger sequencing was used.

**Fig. 2:**
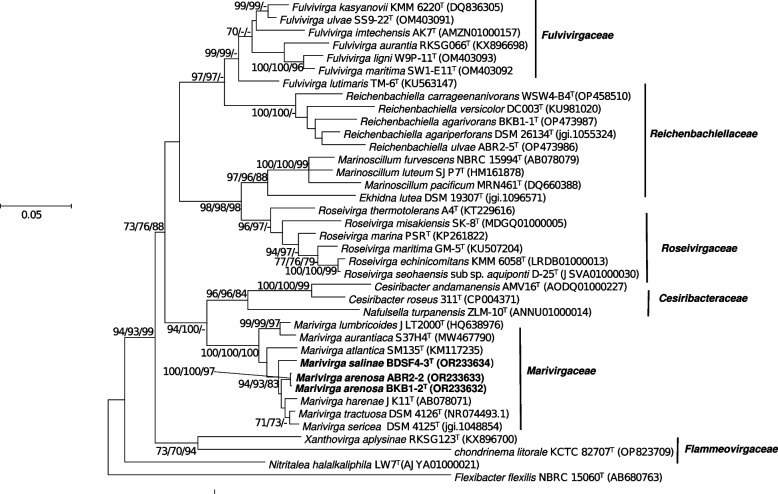
Maximum likelihood (ML) tree based on 16S rRNA gene sequences showing the phylogenetic positions of the solates. ML tree was made using the 16S rRNA sequence to illustrate the phylogenetic connections between the isolates and the type species of different genus within the order *Cytophagales*. Bootstrap values (> 70%) are indicated at the branch points ML/NJ/MP, based on 1000 replication methods. *Flexibacter flexilis* NBRC 15060^ T^ was applied as an outgroup

The phylogenetic trees were constructed using 16S rRNA genes to find the position of new isolates in the family *Marivirgaceae*. The phylogenetic trees showed a monophyletic clustering of strains BKB1-2^ T^, ABR2-2, and BDSF4-3^ T^ with *M. lumbricoides* JLT 2000^ T^, *M. aurantiaca* S37H4^T^, *M. atlantica* SM 1354^ T^, *M. harenae* JK11^T^, *M. tractuosa* DSM 4126^ T^, and *M. sericea* DSM 4125^ T^ (Fig. 2). Based on the 16S rRNA similarity values and the phylogenetic trees, strains *M. tractuosa* KCTC 2958^ T^, *M. harenae* KCTC 92433^ T^, *M. sericea* KCTC 2899^ T^, *M. atlantica* KCTC 42392^ T^*,* and *M. lumbricoides* KCTC 92621^ T^ were selected as reference strains for further taxonomic study. The sequences of the 16S rRNA gene of the strains BKB1-2^ T^, ABR2-2, and BDSF4-3^ T^ were submitted to GenBank/EMBL/DDBJ having accession numbers of OR233632, OR233633, and OR233634.

### Physiological, biochemical, and chemotaxonomic characterization

All three isolated strains were Gram-stain-negative, rod-shaped, strictly aerobic, and exhibited gliding motility. All three isolates exhibited optimal growth within the temperature range of 25–30 °C, an optimal NaCl concentration range of 2–4% (w/v), and an optimum pH range of 6.5–7.5. The comprehensive physiological properties of the isolated strains and the existing species in the genus *Marivirga* are summarized in Table 1.

**Table 1:** Differential physiological properties of three isolates and the reference species within the genus *Marivirga.* Strains: 1, BKB1-2^ T^; 2, ABR2-2; 3, BDSF4-3^ T^; 4, *M. tractuosa* DSM 4126^ T^; 5, *M. harenae* KCTC 92433^ T^; 6, *M. sericea* DSM 4125^ T^; 7, *M. atlantica* SM 1354^ T^; 8, *M. lumbricoides* CGMCC 1.10832^ T^

Characteristics	1	2	3	4	5	6	7	8
Cell shape	Rod	Rod	Rod	slender rod	slender rod	slender rod	rod	rod
Colony morphology	circular dark orange, smooth	circular dark orange, smooth	circular light orange, smooth	circular shiny	circular orange smooth	circular shiny dark orange	orange- circular convex	orange, oval, glossy opaque
Length & width (μm)	2.4–3.7 0.2–0.3	3.5–3.6 0.2–0.3	2.9–3.1 0.2–0.3	10–50 0.4–0.5	2.5–17 0.4–0.6	30–100 0.4–0.5	2.0–8.0 0.2–0.4	1.7–10 0.3–0.4
Range of NaCl tolerance (optimal) (% w/v)	1–10 (2–4)	1–10 (2–4)	1–9 (2–4)	3–10 (2–4)	5–10 (3–5)	5–10 (3–5)	0.5–11 (3)	0–15 (3–5)
Range of temperature (optimal) (℃)	10–37 (25–30)	10–37 (25–30)	10–37 (25–30)	5–35 (25–30)	5–35 (25–30)	5–38 (25–30)	4–40 (25–30)	4–40 (25–30)
Range of pH (optimal)	6–10 (6.5–7.5)	6–10 (6.5–7.5)	6.5–9.5 (6.5–7.5)	NA	6–9 (7.0)	NA	5.5–8.5 (6–7)	5.0–8.0 (6–7)

All results were obtained from this study except for the reference strains from the literatures [14–17]

*NA* not available

The strains BKB1-2^ T^, ABR2-2, and BDSF4-3^ T^ hydrolyzed Tweens 20 and 40, and casein but could not hydrolyze Tween 80. No strains produced flexirubin-type pigments. In the API ZYM kit, all three isolates and reference strains were positive for the activities of acid phosphatase, alkaline phosphatase, cystine arylamidase, α-chymotrypsin, esterase (C4), esterase lipase (C8), lipase (C14), leucine arylamidase, naphthol-AS-BI-phosphohydrolase, trypsin, and valine arylamidase, while negative for the activities of β-glucuronidase, α-fucosidase, and α-mannosidase. Among all the newly isolated strains and the reference strains, only *M. lumbricoides* KCTC 92621^ T^ showed α-galactosidase and β-galactosidase activities. Furthermore, all strains were positive for β-glucosidase except *M. harenae* KCTC 92433^ T^. In API 50CH, all three strains utilized only esculin ferric citrate and potassium 5-ketogluconate. Moreover, in GEN III MicroPlates (Biolog), all three strains utilized acetic acid, α-D-glucose, D-maltose, sodium butyrate, and D-trehalose. Among the tested *Marivirga* strains, only strain BKB1-2^ T^ utilized D-malic acid and bromo succinic acid. Additionally, strains BKB1-2^ T^ and ABR2-2 exhibited a positive reaction to 1% sodium lactate and the three isolated strains could not utilize formic acid. Despite that the strains share certain biochemical characteristics, there were notable differences in various biochemical tests between the isolates and the reference strains, as shown in Table 2.

**Table 2:** Differential biochemical characteristics of three isolates and the reference species within the genus *Marivirga*

Biochemical tests	1	2	3	4	5	6	7	8
**Enzyme activity (API ZYM)**								
* N-*Acetyl*- β-*glucosaminidase	-	-	-	-	-	-	-	+
* α*-Galactosidase	-	-	-	-	-	-	W	+
* β-*Galactosidase	-	-	-	-	-	-	-	+
* α-*Glucosidase	-	-	-	+	-	+	+	+
* β-*Glucosidase	+	+	+	+	-	+	+	+
**API 50 CH acid production**								
L-Arabinose	-	-	-	-	-	-	-	+
Arbutin	-	-	-	-	-	-	-	+
D-Lactose	-	-	-	-	W	-	-	+
D-Melezitose	-	-	-	W	-	-	-	+
Potassium 5-ketogluconate	+	+	+	-	+	-	+	-
D-Turanose	-	-	-	W	-	-	-	+
**Biolog GEN III**								
L-Arginine	+	-	-	+	-	-	+	-
L-Aspartic acid	-	+	+	-	-	-	-	+
D-Arabitol	-	+	-	-	+	-	-	-
Acetoacetic acid	-	+	+	+	-	-	+	+
Acetic acid	+	+	+	+	-	+	+	+
Aztreonam	-	+	+	+	-	-	-	-
Bromo-succinic acid	+	-	-	-	-	-	-	-
D-Cellobiose	-	+	+	+	-	-	+	-
Formic acid	-	-	-	+	-	-	-	-
* α-*D-Glucose	+	+	+	+	-	-	+	+
Gelatin	-	+	-	-	-	-	-	+
Glycyl-l-proline	-	+	+	+	-	-	+	+
L-Glutamic acid	+	+	-	-	-	-	-	+
α-Keto-glutaric acid	-	+	-	-	-	-	-	-
D-Maltose	+	+	+	+	+	-	+	+
D-Mannose	+	-	+	-	+	-	+	+
D-Malic acid	+	-	-	-	-	-	-	-
L-Malic acid	+	-	-	-	-	-	-	+
Propionic acid	-	-	+	-	-	-	+	-
Pectin	+	-	-	-	-	-	-	+
Sucrose	-	+	-	+	-	+	-	-
D-Salicin	-	-	+	+	-	-	-	-
1% Sodium lactate	+	+	-	-	-	-	-	-
D-Trehalose	+	+	+	+	-	-	+	+
**Nitrogen metabolism**								
DNRA	-	-	+	+	+	-	-	-
Nitrite reduction	-	-	+	+	+	-	-	-

Strains: 1, BKB1-2^ T^; 2, ABR2-2; 3, BDSF4-3^ T^; 4, *M. tractuosa* KCTC 2958^ T^; 5, *M. harenae* KCTC 92433^ T^; 6, *M. sericea* KCTC 2899^ T^; 7, *M. atlantica* KCTC 42392^ T^; 8*, M. lumbricoides* KCTC 92621^ T^. + , Positive; -, negative; w, weak

For the chemotaxonomic characterization, the most abundant fatty acids detected in the three newly isolated strains and reference strains were iso-C_15:0_ and C_15:1_ G, which ranged from 19.1–40.8% and 15.2–20.5%, respectively. Among the three isolated strains, strain BKB1-2^ T^ additionally had summed feature 3 (C_16:1_ ω7c/C_16:1_ ω6c) at a level of 20.8% as a prominent fatty acid. Although the fatty acid profiles among isolated strains and the existing species were similar, there were differences among all eight strains (Table S2). All three strains had menaquinone 7 (MK-7) as a respiratory quinone, which is consistent within the genus *Marivirga*. The polar lipid profiles of the three isolated strains were composed of phosphatidylethanolamine (PE), unidentified amino lipids (AL), and unidentified lipids (L). The strain BKB1-2^ T^ had PE, one AL, and five unidentified lipids L1-L5, while the strain ABR2-2 had PE, one AL, and six unidentified lipids L1-L6. The strain BDSF4-3^ T^ had PE, two AL1-AL2, and six unidentified lipids L1-L6 (Fig S1).

### Genome sequencing, analysis and phylogeny

The complete genomes of the strains BKB1-2^ T^, ABR2-2, and BDSF4-3^ T^ and *M. harenae* KCTC 92433^ T^ were sequenced using Oxford Nanopore Technology (ONT). All genomes were assembled and checked for completeness and contamination. The CheckM values of the determined genomes were 99.7–100, showing that all genome sequences were obtained with a high quality. The genomes of strains ABR2-2 and BDSF4-3^ T^ were assembled into a single contig, each formed a circular chromosome with sizes of 3.94 and 4.49 Mb, respectively. In contrast, the genome of strain BKB1-2^ T^ consisted of two contigs: contig 1 formed a circular chromosome with a size of 4.02 Mb, while contig 2 was a plasmid 25,320 bp long with a G + C content of 35.5%. Overall, the G + C content in the genomes of the three newly isolated strains ranged from 33.7–34.5%, aligning with the genus *Marivirga*. The PGAP annotation from NCBI provided information on the total number of genes, coding sequences (CDS), tRNAs, and rRNAs in the genome, and this is summarized in Table 3. The genomes of the strains BKB1-2^ T^, ABR2-2, and BDSF4-3^ T^ and *M. harenae* KCTC 92433^ T^ were uploaded to NCBI with GenBank accession numbers CP129968, CP129970, CP129971, and CP130565, respectively.

**Table 3:** Genomic properties of three isolates and the reference strains within the genus *Marivirga*

Genomes properties	1	2	3	4	5	6	7	8	9
Accession no	CP129968	CP129970	CP129971	GCF_000183425	CP130565	GCF_900177665	GCF_016734805	GCF_014636295	GCA_016734805
Genome size (Mbp)	4.02	3.94	4.49	4.51	4.34	4.74	4.19	5.93	4.2
G + C content (%)	33.7	33.7	34.5	35.5	35.4	36	36.6	36.8	36.5
Total number of genes	3471	3417	3807	3800	3738	4082	3630	4978	3628
Total number of proteins	3417	3363	3743	3745	3659	4011	3542	4906	3582
rRNAs	6	6	6	6	6	7	3	3	3
tRNAs	38	39	39	38	39	38	36	39	36
ncRNAs	3	3	3	3	3	2	3	3	3
Pseudogene	7	7	16	16	31	23	46	27	7

Strains: 1, BKB1-2^ T^; 2, ABR2-2; 3, BDSF4-3^ T^; 4, *M. tractuosa* DSM 4126^ T^; 5, *M. harenae* JK11^T^; 6, *M. sericea* DSM 4125^ T^; 7, *M.* *atlantica* SM 1354^ T^; 8, *M. lumbricoides* CGMCC 1.10832^ T^; 9, *M. aurantiaca* S37H4^T^

**Fig. 3:**
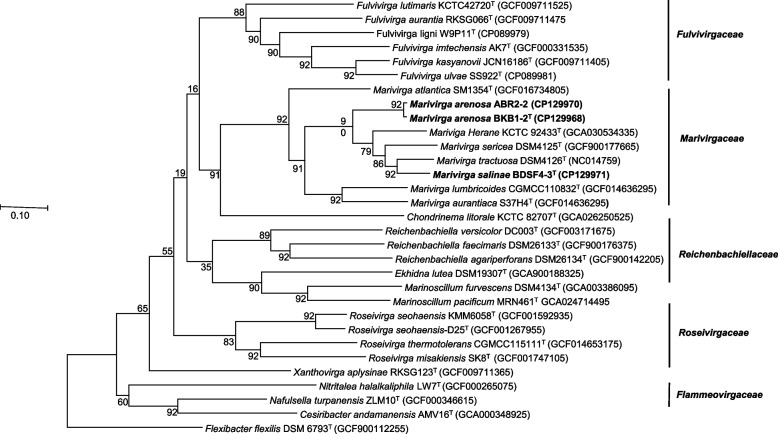
Maximum-likelihood (ML) tree based on 92 core genes using the UBCG pipeline. ML tree were constructed from 92 core genes through the UBCG pipeline, confirming the relationships among the three isolates and the existing strains of the genus *Marivirga*. *Flexibacter flexilis* DSM 6793^ T^ (GCF900112255) was used as an outgroup. The branch nodes are labeled with bootstrap values based on 1000 replicates, and the scale bar represents 0.1 substitutions per site

The ANI and dDDH values between the two novel isolates BKB1-2^ T^ and BDSF4-3^ T^ and the existing type strains within the genus *Marivirga* ranged from 70.5–76.5% and 18.1–25.7%, respectively. The ANI and dDDH values fell below the specified thresholds for species differentiation, which are typically 95–96% for ANI and 70% for dDDH [41, 56]. Among the three newly isolated strains, the ANI and dDDH values between the isolates BKB1-2^ T^ and ABR2-2 were 97.8% and 79.5%, respectively (Table 4), which were higher than the threshold values. Thus, based on ANI and dDDH calculations, only the strains BKB1-2^ T^ and BDSF4-3^ T^ could be considered novel species. A genome-based phylogenetic tree showed similar monophyletic clustering of the three strains with *M. atlantica* SM1354^T^, *M. harenae* JK11^T^, *M. sericea* DSM4125^T^, *M. tractuosa* DSM 4126^ T^, *M. lumbricoides* JLT2000^T^, and *M. aurantiaca* S37H4^T^, which was also observed in the 16S rRNA gene-based trees (Fig. 3).

**Table 4:**
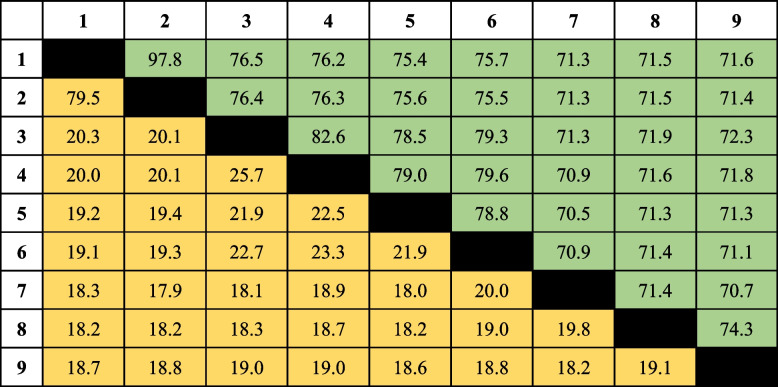
ANI (upper triangle in green) and dDDH (lower triangle in yellow) between three isolates and the reference strains within the genus Marivirga

Strains: 1, BKB1-2^ T^; 2, ABR2-2; 3, BDSF4-3^ T^; 4, *M. tractuosa* DSM 4126^ T^; 5, *M. harenae* JK11^T^; 6, *M. sericea* DSM 4125^ T^; 7, *M.* *atlantica* SM 1354^ T^; 8, *M. lumbricoides* CGMCC 1.10832^ T^; 9, *M. aurantiaca* S37H4^T^

### Comparative genomes functional analysis

#### Metabolic pathways analysis using KEGG and RAST servers

**Fig. 4:**
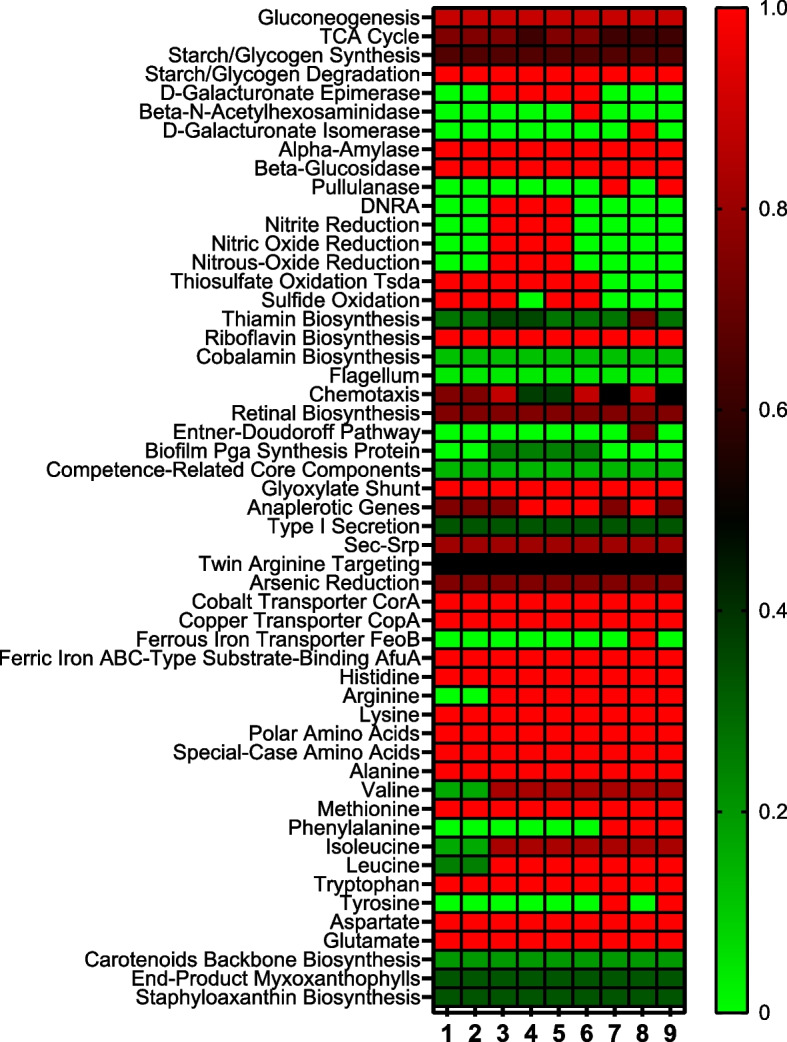
Heatmap showing the distribution of various metabolic pathways identified through KEGG analysis among the *Marivirga* strains. The scale bar indicates that the intensity of color reflects the completeness of pathways

The genomes of three new isolates and the reference strains were analyzed for metabolic pathways using KEGG and RAST servers. The metabolic pathways were constructed using the KEGG database and then plotted in a heatmap (Fig. 4). The heatmap showed that all strains in the genus *Marivirga* possessed central metabolic pathways such as aerobic respiration, sugar metabolism, and various amino acid biosynthesis pathways. The KEGG analysis emphasized that all strains in the genus *Marivirga* have the potential to synthesize certain essential amino acids including histidine, lysine, methionine, threonine, and tryptophan and non-essential amino acids including alanine, aspartate, asparagine, cysteine, glycine, glutamine, proline, and serine. Among three isolated strains, only strain BDSF4-3^ T^ had a complete pathway for the synthesis of arginine and leucine and partial pathways for valine and isoleucine. Furthermore, all the *Marivirga* species possessed a complete pathway for the production of riboflavin and additional partial pathways for the synthesis of thiamin and cobalamin (Fig. 4). The KEGG pathways also showed that strains in the genus *Marivirga* contained genes of various hydrolytic enzymes. All strains harbored genes for the synthesis of α-amylase and β-glucosidase. Strain BDSF4-3^ T^ harbored genes for D-galacturonate epimerase. Among the reference strains, *M. lumbricoides* CGMCC 1.10832^ T^ carried genes for D-galacturonate isomerase and *M. sericea* DSM 4125^ T^ had genes for β-N-acetyl hexosaminidase, while *M. atlantica* SM 1354^ T^ and *M. aurantiaca* S37H4^T^ carried genes for the production of pullulanase [57]. These enzymes have diverse applications across multiple sectors, including food, pharmaceutical industries, biofuel production, and waste treatment [58, 59].

The KEGG analysis further highlighted the role of *Marivirga* species in the nitrogen cycle of coastal ecosystems. Strains BDSF4-3^ T^, *M. tractuosa* DSM 4126^ T^, and *M. harenae* KCTC 92433^ T^ had a completed pathway of DNRA and reduction of nitrite, nitric oxide, and nitrous oxide (Fig. 4). The in vitro investigation of DNRA activity was conducted using an MQuant colorimetric strip to determine ammonium production, providing additional support to the genome analysis. The results showed strong activities of ammonium production from nitrate for the strains BDSF4-3^ T^, *M. tractuosa* DSM 4126^ T^, and *M. harenae* KCTC 92433^ T^ (Table 2). Second to DNRA, the reduction of nitrite was determined using Griess reagent. During incubation, the strains reduced nitrite, resulting in a decrease in its concentration in the media. The assay showed that strain BDSF4-3^ T^, *M. tractuosa* DSM 4126^ T^, and *M. harenae* KCTC 92433^ T^ exhibited ability for nitrite reduction (Table 2). Microbes utilize the DNRA pathway to transform nitrate into ammonium, effectively preserving bioavailable nitrogen within the ecosystem [60]. DNRA occurs in various ecosystems, including estuary and Antarctica [61]. This pathway not only aids in preserving nitrogen within these ecosystems but also plays a role in reducing the emission of harmful greenhouse gases, such as nitrous oxide [62]. Additionally, the reduction of nitrite and nitrate by marine microbes further is important for marine ecosystems and for global nitrogen cycling [34].

Based on RAST annotation, the genomes of strains BKB1-2^ T^ and ABR2-2 showed the presence of 243 and 244 functional sub systems, while the strain BDSF4-3^ T^ had 247 subsystems. In the RAST analysis, the largest number of annotated genes were assigned to amino acids and derivatives, which ranged from (209–257 genes), carbohydrates (102–193 genes), and protein metabolism (154–171 genes). Furthermore, the RAST analysis showed that all the isolates and reference strains in the genus *Marivirga* carried genes for the metabolism of nitrogen (7–34 genes), potassium (11–14 genes), phosphorus (14–25 genes), and sulfur (7–11 genes). Moreover, the RAST system identified 22–29 stress response genes that are essential for the protection of bacteria from reactive oxygen species in the marine environment (Table 5).

**Table 5:** Overview of the RAST subsystem and the numbers of genes involved in each metabolism of three isolates and reference strains within the genus *Marivirga*

RAST subsystem category	1	2	3	4	5	6	7	8	9
Co-factors, vitamins, prosthetic groups, pigments	128	127	133	138	133	135	130	149	142
Cell wall and capsule	26	27	22	25	27	28	27	27	22
Virulence, disease, and defense	28	33	36	43	36	38	45	50	36
Potassium metabolism	12	11	12	12	14	12	13	12	13
Miscellaneous	12	12	11	12	13	14	11	12	16
Membrane transport	6	6	11	18	7	12	12	51	11
Iron acquisition & metabolism	0	0	0	0	0	0	0	3	0
RNA metabolism	34	34	38	38	40	39	33	39	36
Nucleosides and nucleotides	53	52	54	55	54	52	55	57	56
Protein metabolism	156	157	157	161	169	168	154	171	162
Cell division and cell cycle	3	3	3	3	3	3	3	3	3
Regulation and cell signaling	7	4	2	13	8	3	7	17	2
Secondary metabolism	10	8	7	7	7	7	7	8	8
DNA metabolism	55	58	62	67	61	60	65	83	75
Fatty acids, lipids, isoprenoids	31	31	31	33	33	34	31	30	31
Nitrogen metabolism	7	7	33	34	32	8	10	9	33
Dormancy and sporulation	1	1	1	1	1	1	1	1	1
Respiration	41	42	40	33	43	38	31	33	33
Stress response	22	23	26	26	23	26	23	29	20
Metabolism aromatic compound	11	11	11	9	9	12	11	11	9
Amino acids and derivatives	209	209	238	257	256	244	225	256	241
Sulfur metabolism	7	7	8	8	9	8	9	11	8
Phosphorus metabolism	14	14	14	14	15	14	19	25	15
Carbohydrates	130	130	135	105	106	146	102	193	15

Strains: 1, BKB1-2^ T^; 2, ABR2-2; 3, BDSF4-3^ T^; 4, *M. tractuosa* DSM 4126^ T^; 5, *M. harenae* JK11^T^; 6, *M. sericea* DSM 4125^ T^; 7, *M.* *atlantica* SM 1354^ T^; 8, *M. lumbricoides* CGMCC 1.10832^ T^; 9, *M. aurantiaca* S37H4^T^

#### Detection of biosynthetic gene clusters (BGCs) using antiSMASH server

The antiSMASH analysis found type III polyketide synthases (PKS) and the terpene classes of BGCs in the genome of the isolates and the type strains in the genus *Marivirga*. Notably, strain BKB1-2^ T^ had an additional BGC for lanthipeptide class I. Compared to the three newly isolated strains, the strain *M. sericea* DSM 4125^ T^ had additional ribosomally synthesized and post-translationally modified peptides (RiPP-like) BGCs, while the strain *M. lumbricoides* CGMCC 1.10832^ T^ had type I PKS, resorcinol, and non-ribosomal peptide synthetase (NRPS-like) BGCs (Table S3). Polyketide synthases constitute a group of enzymes that play a role in the synthesis of polyketides, which have antimicrobial properties [63]. Meanwhile, lanthipeptide class I refer to ribosomally synthesized antibacterial molecules, which are often referred to as lantibiotics. These bioactive compounds have potential applications in the food industry and can be used against highly antibiotic resistant pathogens [64].

#### Detection of carbohydrate-active enzymes (CAZymes) using dbCAN server

The genomes of three newly isolated strains and six type strains in the genus *Marivirga* were analyzed for CAZyme gene clusters (CGCs) using the dbCAN database. The dbCAN analysis showed that the genome of strain BKB1-2^ T^ harbors a total of 53 CAZymes, distributed into 28 glycosyltransferases (GTs), 17 glycoside hydrolases (GHs), four carbohydrate esterases (CEs), three auxiliary activities (AAs), and one carbohydrate binding module (CBM). The strain ABR2-2 had a total of 56 CAZyme distributed into 27 GTs, 21 GHs, three CEs, three AAs, and two CBMs, while the strain BDSF4-3^ T^ had a total of 59 CAZyme distributed into 35 GTs, 16 GHs, three CEs, one AA, and three CBMs. Among three new isolates and existing strains in the genus *Marivirga*, strain *M. lumbricoides* CGMCC 1.10832^ T^ had the highest CAZymes of 152, distributed into 48 GTs, 75 GHs, 14 CEs, four AAs, three polysaccharide lyases (PLs), and three CBMs (Table 6). Furthermore, the percentage of CAZymes and ratio of GH per Mb of genome were calculated for all the *Marivirga* strains. The percentage of CAZymes out of the total genes was 1.52, 1.64, and 1.55% for the strains BKB1-2^ T^, ABR2-2, and BDSF4-3^ T^, respectively. The percentage of CAZymes was 3.05 for the strain *M. lumbricoides* JCM 18012^ T^, which was higher in the genus *Marivirga*. Furthermore, the GHs per Mbp in the genomes of strains BKB1-2^ T^, ABR2-2, and BDSF4-3^ T^ were 4.23, 5.33, and 3.56, respectively (Table S4). The number of CAZymes in the family *Marivirgaceae* (53–152 CAZymes) closely resembled that of neighboring families within the phylum *Bacteroidota,* including *Reichenbachiellaceae* (79–216 CAZymes) [49], *Fulvivirgaceae* (55–264 CAZymes) [19], and *Flavobacteriaceae Zobellia* sp (257–315 CAZymes) [65, 66]. The presence of high numbers of CAZymes in bacteria offers insights into their ecological roles, adaptability, and potential applications across various biotechnological processes [67].

**Table 6:** Determination of CAZymes in three isolates and reference strains within the genus *Marivirga*, using dbCAN2

CAZyme	1	2	3	4	5	6	7	8	9
CBM	1	2	3	3	3	3	3	8	2
AA	3	3	1	2	2	4	1	4	3
CE	4	3	3	3	3	3	4	14	4
GH	17	21	16	25	22	25	30	75	28
GT	28	27	35	32	32	37	32	48	37
PL	0	0	1	0	0	0	0	3	0
Total	53	56	59	65	62	72	70	152	74

Strains: 1, BKB1-2^ T^; 2, ABR2-2; 3, BDSF4-3^ T^; 4, *M. tractuosa* DSM 4126^ T^; 5, *M. harenae* JK11^T^; 6, *M. sericea* DSM 4125^ T^; 7, *M.* *atlantica* SM 1354^ T^; 8, *M. lumbricoides* CGMCC 1.10832^ T^; 9, *M. aurantiaca* S37H4^T^

*CBM* carbohydrate-binding modules, *AA* auxiliary activities, *CE* carbohydrate esterase, *GH* glycoside hydrolase, *GT* glycosyltransferase, *PL* polysaccharide lyase

#### Detection of heavy metal metabolism genes using the KEGG database

The KEGG pathways showed that three isolates and the reference strains had pathways for reduction of arsenic and also carried transporter genes including *CorA, CopA,* and *AfuA* for transport of cobalt, copper, and ferric iron into the cell, respectively **(**Fig. 4**)** [68]. The *CorA* protein is typically known for its role in transporting magnesium ions, but some members of the *CorA* family could also facilitate the transportation of cobalt (Co^2+^) and nickel (Ni^2+^) [69]. *CopA* is the most important transporter protein for the transport of metals in microbes. The *CopA* functions by binding to two Cu^2+^ ions and thereafter transferring them to the periplasmic space with the help of the Cu chaperone (*CusF*) using ATP as an energy source [68]. Furthermore, the isolates and the type strains in the genus *Marivirga* carried *AfuA*, which is an ABC-type transporter responsible for ferric iron transport. Among all strains, only *M. lumbricoides* JCM 18012^ T^ carried *FeoB*, a cytoplasmic membrane transporter protein that participated in ferrous iron transport [70, 71].

#### In vitro polysaccharide degradation

The degradation of complex polysaccharides, including agar, alginate, cellulose, chitin, κ-carrageenan, λ-carrageenan, ι-carrageenan, laminarin, starch, and xylan, was tested in both solid and liquid media. First, the degradation of polysaccharides was assessed on solid media by identifying a clear zone around the colonies. The degradation of these complex polysaccharides was further tested by detecting reducing sugar using the 3, 5-dinitrosalicylic acid assay. The results showed the strain BKB1-2^ T^ degraded cellulose, chitin, laminarin, and starch, the strain ABR2-2 degraded chitin, laminarin, and starch, and the strain BDSF4-3^ T^ degraded chitin, laminarin, starch, and xylan (Table S5).

The in vitro degradation was supported by the identification of CAZymes genes within the genome. The breakdown of starch was facilitated by the existence of glycoside hydrolase families GH13 and GH16, primarily characterized as α-amylases [72]. All strains degraded chitin and carried GH23 that may involve in the degradation of chitin [73]. The hydrolysis of laminarin and xylan was facilitated by GH16 and GH3 in the genome, respectively (http://www.cazy.org/GH16.html) (**Table S5**). Oligosaccharides generated from the breakdown of polysaccharides had been studied to exhibit diverse biological activities, rendering them suitable for use in the cosmetic, functional food, and medical industries [74, 75]. The decomposition of laminarin, a highly abundant marine polysaccharide, plays a significant role in the turnover of marine polysaccharides [8]. Furthermore, the degradation of xylan holds significant importance for the biofuel, animal feed, pulp, and paper sectors [76]. Overall, the analyses emphasize the significance of these strains in their capacity to degrade complex polysaccharides, underscoring their importance in the carbon cycle and in various biotechnological applications.

#### Determination of the minimal inhibitory concentration (MIC) of heavy metals for the isolated strains

All three newly isolated strains were examined for their tolerance to various heavy metals. The MIC values of the heavy metals, including Cu^2+^, Co^2+^, Ni^2+^, Zn^2+^, Mn^2+^, and Hg^2+^ were different for the three isolates. Strain ABR2-2 tolerated high concentrations of various heavy metals compared to other strains. Strain ABR2-2 exhibited tolerance to heavy metals with minimum inhibitory concentrations of Co^2+^ (6 mM), Ni^2+^ (5 mM), Zn^2+^ (4 mM), Mn^2+^ (50 mM), and Hg^2+^ (0.4 mM). Strain BKB1-2^ T^ had MIC to Co^2+^ (6 mM), Ni^2+^ (5 mM), Zn^2+^ (2 mM), Mn^2+^ (50 mM), and Hg^2+^ (0.2 mM), while strain BDSF4-3^ T^ had MICs of Co^2+^ (3 mM), Ni^2+^ (3 mM), Zn^2+^ (2 mM), Mn^2+^ (30 mM), and Hg^2+^ (0.4 mM) (Table 7). Strains capable of growing in the presence of 0.5 mM of Co^2+^, Cu^2+^, Ni^2+^_,_ Zn^2+^, and 0.05 mM of Hg^2+^ were classified as tolerant strains [52, 53]. The results are significant and comparable to those of known metal-resistant strains isolated from the deep sea. For example, *Dietzia maris* and *Pseudoalteromonas shioyasakiensis* strains, isolated from deep-sea sediments, demonstrated resistance to a range of heavy metals. *Dietzia maris* showed MICs of Cu^2+^ (2.8–5.7 mM), Co^2+^ (0.6 mM), Ni^2+^ (3.0 mM), Zn^2+^ (2.2 mM), and Mn^2+^ (14.3 mM) [77]. On the other hand, *Pseudoalteromonas shioyasakiensis*, exhibited MICs of Cu^2+^ (5.7 mM), Co^2+^ (0.3–0.6 mM), Ni^2+^ (3.0–3.6 mM), and Zn^2+^ (0.8–8.5 mM) [77]. Furthermore, the well-known metal-resistant bacteria species *Cupriavidus metallidurans* MSR33 showed elevated MIC values for Cu^2+^ (3.8 mM), Co^2+^ (20 mM), Ni^2+^ (6.0 mM), Zn^2+^ (17 mM), and Hg^2+^ (0.1 mM) [78, 79] (Table 7).

**Table 7:** Minimum inhibitory concentration of heavy metals for newly isolated strains

Strains	MIC (mM)					
	**Cu**^**2+**^	**Co**^**2+**^	**Ni**^**2+**^	**Zn**^**2+**^	**Mn**^**2+**^	**Hg**^**2+**^
**BKB1-2**^**T**^	0.2	6	5	2	50	0.2
**ABR2-2**	0.4	6	5	4	50	0.4
**BDSF4-3**^**T**^	0.2	3	3	2	30	0.4
^***a***^*** Dietzia maris***	2.8–5.7	0.6	3.0	2.2	14.3	NA
^***b***^*** P. shioyasakiensis***	5.7	0.6	3.0–3.6	0.8–8.5	NA	NA
^***c***^***C. metallidurans* MSR33**	3.8	20	6	17	NA	0.1

*NA* not available

^a^*Dietzia maris* [77]

^b^*Pseudoalteromonas shioyasakiensis* [77]

^c^*Cupriavidus metallidurans* MSR33 [79]

These results were further supported by the detection of heavy metal transporter genes in the genomes of *Marivirga* strains. All the strains carried a *CorA* transporter protein involved in the transport of (Co^2+^) and nickel (Ni^2+^). Additionally, the *CopA* genes are prevalent in contaminated environments and participate not only in the metabolism of Cu^2+^ but also in handling other heavy metal, including Ni^2+^, Hg^2+^, and CrO_4_^2−^ [52]. These analyses highlight the importance of *Marivirga* strains in terms of heavy metal remediation in marine environments.

#### Description of *Marivirga arenosa* sp. nov.

Arenosa (a.re.no’sa. L. fem. adj. arenosa, sandy) referring to the source of isolation sea sand.

The cells are Gram negative bacilli, strictly aerobic, and have gliding motility. Produces a dark orange, circular, smooth colony on MA. The optimum growth occurs at temperatures 25–30 °C, pH 6.5–7.5, and NaCl 2–4% (w/v). The strain is positive for the activities of acid phosphatase, alkaline phosphatase, cystine arylamidase, α-chymotrypsin, esterase (C4), esterase lipase (C8), lipase (C14), leucine arylamidase, naphthol-AS-BI-phosphohydrolase, trypsin, and valine arylamidase. The strain shows a positive reaction in Biolog GEN III plate for acetic acid, L-arginine, α-D-glucose, D-galactose, L-glutamic acid, D-maltose, D-mannose, D-malic Acid, L-malic Acid, sodium butyrate, and D-trehalose. Moreover, the strain can hydrolyze casein, cellulose, chitin, laminarin, starch, Tween 20, and Tween 40. The major fatty acids are iso-C_15:0_ and summed feature 3 (C_16:1_ ω7c/C_16:1_ ω6c), while the main polar lipids and the respiratory quinone are phosphatidylethanolamine and menaquinone-7. The genomic DNA G + C content of the type strain is 33.7%.

The type strain, BKB1-2^ T^ (= KCTC 82989^ T^ = InaCC B1618^T^) was isolated from the sea sand collected from the West Sea, Republic of Korea.

#### Description of *Marivirga salinae* sp. nov.

Salinae*. (L. Sal.* is derived from the Latin word *sal* meaning salt), referring to to the source of isolation at a salt farm.

The cells are Gram negative bacilli, strictly aerobic, and have gliding motility. Produces a light orange, circular, smooth colony on MA. The optimum growth occurs at temperatures 25–30 °C, pH 6.5–7.5, and NaCl 2–4% (w/v). The strain is positive for the activities of acid phosphatase, alkaline phosphatase, cystine arylamidase, α-chymotrypsin, esterase (C4), esterase lipase (C8), lipase (C14), leucine arylamidase, naphthol-AS-BI-phosphohydrolase, trypsin, and valine arylamidase. The strain shows a positive reaction in Biolog GEN III plate for acetic acid, acetoacetic acid, L-aspartic acid, D-cellobiose, gentiobiose, α-D-glucose, D-maltose, D-mannose, propionic acid, sodium butyrate, D-salicin, and D-trehalose. The strain can hydrolyze casein, chitin, laminarin, starch, Tween 20, Tween 40, and xylan. The predominant fatty acids are iso-C_15:0_ and iso-C_15:1_ G while the main polar lipids and the respiratory quinone are phosphatidylethanolamine and menaquinone-7, respectively. The genomic DNA G + C content of the type strain is 34.4%.

The type strain, BDSF4-3^ T^ (= KCTC 82973^ T^ = InaCC B1619^T^), was isolated from microbial mat collected from the West Sea, Republic of Korea.

## Conclusion

In conclusion, three strains, BKB1-2^ T^, ABR2-2, and BDSF4-3^ T^, were isolated from sea sand, green alga, and microbial mats collected at the West Sea, Korea. Using a polyphasic taxonomy approach, it was found that the three isolates were affiliated with the genus *Marivirga* within the phylum *Bacteroidota.* Notably, two of them, BKB1-2^ T^ and BDSF4-3^ T^, were considered to represent two novel species in the genus *Marivirga*. Currently, the family *Marinivrgacease* consists of only one genus: *Marivirga*. The comparative genome analysis of the *Marivirgaceae* family highlighted the significance of all strains in terms of complex polysaccharide degradation, heavy metal resistance, the synthesis of various amino acids and vitamins. The isolates possessed important nitrogen metabolic pathways, including DNRA, nitrite, nitric oxide, and nitrous oxide reduction, which collectively contribute to the nitrogen cycle within ecosystems. Furthermore, the in vitro analysis revealed that the newly isolated strains are capable of degrading complex polysaccharides, including chitin, cellulose, laminarin, starch, and xylan, which is crucial for the carbon cycle. Additionally, these strains displayed tolerance to heavy metals, including Co^2+^, Ni^2+^, Zn^2+^, Mn^2+^, and Hg^2+^. Our analysis highlights that strains within the genus *Marivirga* hold potential for future utilization in various environmental and industrial sectors.

### Supplementary Information

Supplementary Material 1.

## Data Availability

The datasets generated and analyzed during the current study are available at the National Center for Biotechnology Information (NCBI). The GenBank/EMBL/DDBJ accession numbers for the 16S rRNA gene sequences of strains BKB1-2^T^, ABR2-2, and BDSF4-3^T^ are OR233632, OR233633, and OR233634, respectively, and the genome sequences are CP129968, CP129970, and CP129971, respectively.

## Abbreviations

ANI: Average Nucleotide Identity
dDDH: Digital DNA–DNA hybridization
MA: Marine agar
CAZymes: Carbohydrate-active enzymes
DNRA: Dissimilatory nitrate reduction to ammonium
DNS: Dinitrosalicylic acid
