# Supplementary material for: Comparative genome analysis of the genus Marivirga and proposal of two novel marine species: Marivirga arenosa sp. nov., and Marivirga salinae sp. nov
Source: BMC Microbiol. 2024 Jul 5;24:245. doi: 10.1186/s12866-024-03393-3 (PMC11225308; doi:10.1186/s12866-024-03393-3)
Supplement: Supplementary file 1 — Supplementary Material 1. [file 12866_2024_3393_MOESM1_ESM.docx]

**Comparative genome analysis of the genus *Marivirga* and proposal of two novel marine species: *Marivirga* *arenosa*** **sp. nov., and *Marivirga salinae* sp. nov.**

Neak Muhammad^1, 2^, Forbes Avila^1, 2^, Song-Gun Kim^1, 2^*

^1^Biological Resource Center/Korean Collection for Type Cultures (KCTC), Korea Research Institute of Bioscience and Biotechnology, Jeongeup, Jeonbuk 56212, the Republic of Korea

^2^Department of Environmental Biotechnology, KRIBB School, University of Science and Technology (UST), Daejeon 34113, the Republic of Korea

***Corresponding author:**

**Song-Gun Kim**

Email: sgkim@kribb.re.kr

**Table S1.** Comparison of similarity values (%) of the 16S rRNA gene among the genus *Marivirga.*

Strains: 1, BKB1-2^T^; 2, ABR2-2; 3, BDSF4-3^T^; 4, *M. tractuosa* DSM 4126^T^; 5, *M. harenae* JK11^T^; 6, *M. sericea* DSM 4125^T^; 7, *M. atlantica* SM 1354^T^; 8, *M. lumbricoides* CGMCC 1.10832^T^; 9, *M. aurantiaca* S37H4^T^.

|  | **1** | **2** | **3** | **4** | **5** | **6** | **7** | **8** | **9** |
| --- | --- | --- | --- | --- | --- | --- | --- | --- | --- |
| **1** | 100 | 99.8 | 97.3 | 97.7 | 97.7 | 97.5 | 96.4 | 95.7 | 91.7 |
| **2** | 99.8 | 100 | 97.3 | 97.9 | 97.7 | 97.7 | 96.4 | 95.7 | 92.9 |
| **3** | 97.3 | 97.3 | 100 | 98.6 | 97.9 | 98.6 | 95.7 | 95.6 | 93.2 |
| **4** | 97.7 | 97.7 | 98.6 | 100 | 98.7 | 99 | 96 | 95.7 | 95.2 |
| **5** | 97.7 | 97.7 | 97.9 | 98.7 | 100 | 98.5 | 95.9 | 96.1 | 93.1 |
| **6** | 97.5 | 97.7 | 98.6 | 99 | 98.5 | 100 | 95.9 | 96 | 91.3 |
| **7** | 96.4 | 96.4 | 95.7 | 96 | 95.9 | 95.9 | 100 | 95.8 | 92.8 |
| **8** | 92.5 | 95.7 | 95.6 | 95.7 | 96.1 | 96 | 95.8 | 100 | 93.2 |
| **9** | 91.7 | 92.9 | 93.2 | 95.2 | 93.1 | 91.3 | 92.8 | 93.2 | 100 |

**Table S2**. Cellular fatty acid compositions (%) of three isolated strains and reference strains in genus *Marivirga.*

Strains: 1, BKB1-2^T^; 2, ABR2-2; 3, BDSF4-3^T^; 4, *M. tractuosa* KCCT 2958^T^; 5, *M. harenae* KCTC 92433^T^; 6, *M. sericea* KCTC 2899^T^; 7, *M. atlantica* KCTC 42392^T^; 8, *M. lumbricoides* KCTC 92621^T^.

| **Fatty acid** | **1** |  | **2** | **3** | **4** | **5** | **6** | **7** | **8** |
| --- | --- | --- | --- | --- | --- | --- | --- | --- | --- |
| **Saturated:** |  |  |  |  |  |  |  |  |  |
| C_16;0_ | 2.1 |  | 1.3 | 3.9 |  | 6.6 | 5.9 | 2.0 | 5.9 |
| C_18;0_ |  |  |  | 1.6 |  | 6.6 | 7.6 |  | 2.0 |
| Unsaturated: |  |  |  |  |  |  |  |  |  |
| C_16:1_ ω5c |  |  |  | 1.2 |  | 1.0 |  |  | 2.4 |
| C_18:1_ ω9c |  |  |  | 2.6 |  | 1.6 | 1.5 |  | 1.5 |
| **Branched:** |  |  |  |  |  |  |  |  |  |
| iso-C_11:0_ |  |  |  |  |  |  | 1.8 |  | 1.9 |
| iso-C_13:0_ |  |  | 1.0 |  | 1.3 |  |  |  |  |
| iso-C_14:0_ | 1.8 |  | 1.8 |  |  | 1.2 |  | 1.3 |  |
| iso-C_15:0_ | 21.1 |  | 37.4 | 40.8 | 38.3 | 18.7 | 23.9 | 28.8 | 19.1 |
| iso-C_15:1_ G | 16.3 |  | 16.7 | 19.1 | 20.5 | 15.2 | 18.9 | 17.0 | 18.2 |
| anteiso-C_15:0_ | 1.9 |  | 2.9 | 1.4 |  | 2.4 | 1.0 | 1.5 |  |
| anteiso-C_17:0_ A |  |  |  |  |  | 1.58 |  |  |  |
| iso-C_16:0_ | 4.6 |  | 3.2 | 2.2 | 3.3 | 4.0 | 1.4 | 3.9 | 1.8 |
| iso-C_16:0_ G | 1.3 |  |  |  | 1.0 | 1.6 |  |  |  |
| iso-C_17:0_ |  |  |  | 1.0 |  |  | 1.5 |  |  |
| **Hydroxy:** |  |  |  |  |  |  |  |  |  |
| C_16:0_ 3-OH |  |  |  |  |  |  |  | 1.2 | 3.0 |
| iso-C_15:0_ 3-OH | 6.0 |  | 5.5 | 3.4 | 3.3 | 2.8 | 3.4 | 4.0 | 3.6 |
| iso-C_16:0_ 3-OH | 9.8 |  | 6.0 | 3.3 | 3.8 | 2.3 | 2.9 | 5.0 | 1.7 |
| iso-C_17:0_ 3-OH | 5.9 |  | 5.5 | 9.6 | 14.7 | 10.0 | 14.2 | 12.9 | 13.9 |
| **Summed features: *** |  |  |  |  |  |  |  |  |  |
| 3 | 20.8 |  | 12.9 | 4.6 | 3.2 | 8.1 | 5.7 | 16.6 | 18.9 |
| 8 |  |  |  |  |  | 1.0 | 1.3 |  | 1.1 |

*Summed features are fatty acids that cannot be resolved reliably from another fatty acid using the chromatographic conditions chosen. The MIDI system groups these fatty acids together as one feature with a single percentage of the total. summed feature 3 comprises C_16:1_ ω7c or C_16:1_ ω6c; summed feature 8 comprises C_18:1_ ω6c and/or C_18:1_ ω7c.

**Figure S1.** Two-dimensional thin-layer chromatography illustrating polar lipid profile of three isolated strains. **A,** BKB1-2^T^; **B**, ABR2-2; **C**, BDSF4-3^T^. The plate was sprayed with molybdophosphoric acid for total lipid detection.


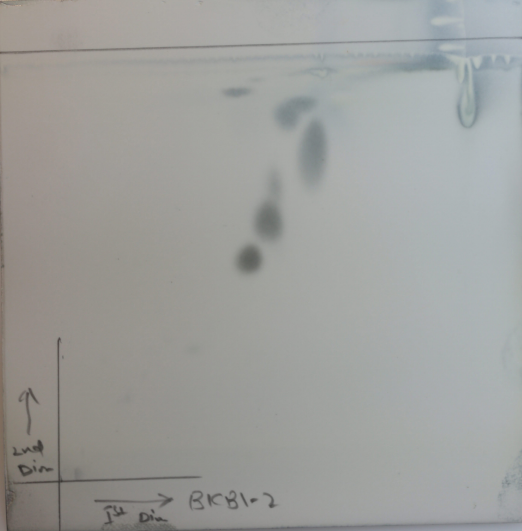


PE

AL

L1

L2

L3

L4

L5


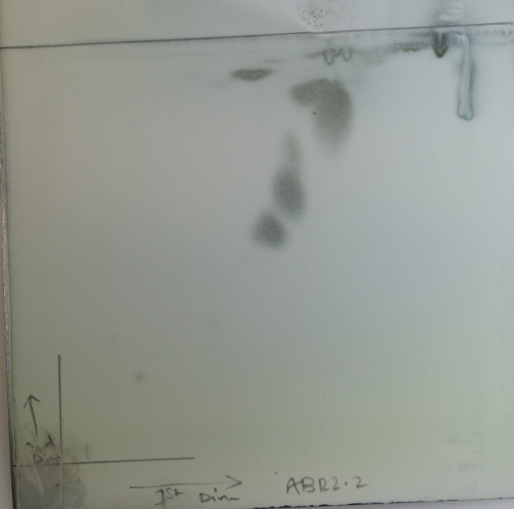


PE

AL

L1

L2

L3

L5

L6

L4

**B**

**A**


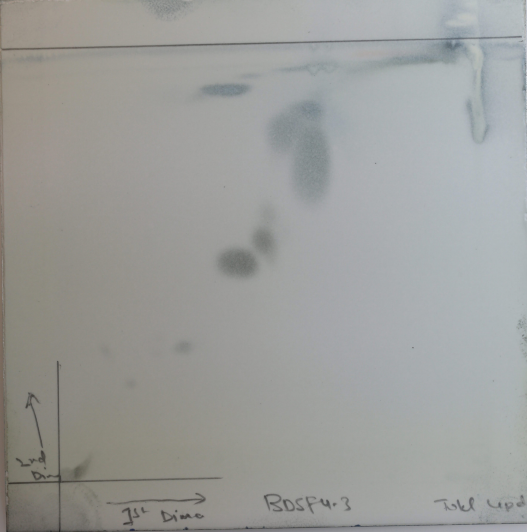


PE

AL1

AL2

L1

L2

L3

L4

L5

L6

**C**

AL, unidentified amino lipid; PE, phosphatidylethanolamine; L, unidentified lipid

**Table S3.** Determination of Biosynthetic gene clusters among three isolates and reference strains within the genus *Marivirga*.

Strains: 1, BKB1-2^T^; 2, ABR2-2; 3, BDSF4-3^T^; 4, *M. tractuosa* DSM 4126^T^; 5, *M. harenae* JK11^T^; 6, *M. sericea* DSM 4125^T^; 7, *M. atlantica* SM 1354^T^; 8, *M. lumbricoides* CGMCC 1.10832^T^; 9, *M. aurantiaca* S37H4^T^. PKS, polyketide synthases; NRPS, non-ribosomal peptide synthetase; RiPP, ribosomally synthesized and post-translationally modified peptides.

| **Strain** | **Type** | ***From** (nt) | ****To** (nt) | **Most similar known cluster** | **Similarity (%)** |
| --- | --- | --- | --- | --- | --- |
| **1** | Type III PKS | 414320 | 455438 |  |  |
|  | Lanthipeptide class I | 1350457 | 1388554 |  |  |
|  | Terpene | 2269904 | 2290800 |  |  |
|  | Terpene | 3442081 | 3462923 | carotenoid, terpene | 57 |
| **2** | Type III PKS | 1463061 | 1504179 |  |  |
|  | Terpene | 2500819 | 2519710 | carotenoid, terpene | 57 |
|  | Terpene | 3577660 | 3598808 |  |  |
| **3** | Type III PKS | 626125 | 667222 |  |  |
|  | Terpene | 1758638 | 1778423 | carotenoid, terpene | 57 |
|  | Terpene | 3028583 | 3049782 |  |  |
| **4** | Terpene | 498343 | 518823 |  |  |
|  | Terpene | 1772773 | 1793615 | carotenoid, terpene | 57 |
|  | Type III PKS | 2627369 | 2668499 |  |  |
| **5** | Type III PKS | 1327752 | 1368840 |  |  |
|  | Terpene | 2512133 | 2531933 | carotenoid, terpene | 57 |
|  | Terpene | 3463727 | 3484875 |  |  |
| **6** | Type III PKS | 321695 | 362783 |  |  |
|  | RiPP-like | 430098 | 443064 |  |  |
|  | Terpene | 109593 | 130435 | carotenoid, terpene | 57 |
|  | Terpene | 817105 | 838250 |  |  |
| **7** | Type III PKS | 466026 | 504910 |  |  |
|  | Type III PKS | 856953 | 898011 |  |  |
|  | Terpene | 912156 | 932998 | carotenoid, terpene | 57 |
|  | Resorcinol | 1540155 | 1582005 |  |  |
|  | Terpene | 934250 | 955428 |  |  |
| **8** | NRPS-like | 605991 | 646534 |  |  |
|  | Type III PKS | 187787 | 230869 |  |  |
|  | Terpene | 214480 | 235322 | carotenoid, terpene | 57 |
|  | Type I PKS | 16100 | 62303 |  |  |
|  | Resorcinol | 1 | 31846 |  |  |
|  | Terpene | 90149 | 110163 |  |  |
|  | Lanthipeptide class I | 31840 | 61577 |  |  |
| **9** | Terpene | 1225254 | 1246417 |  |  |
|  | Arylpolyene | 367935 | 412204 | flexirubin | 16 |
|  | Terpene | 1 | 15329 | carotenoid, terpene | 57 |
|  | Type III PKS | 59034 | 100116 |  |  |

*From indicates the gene's initiation position of biosynthetic gene cluster (BGC)

**To indicates the gene's termination positions of biosynthetic gene cluster (BGC)

**Table S4.** Characteristics of genome of three isolated strains and all reference strains within the genus *Marivirga.*

Strains: 1, BKB1-2^T^; 2, ABR2-2; 3, BDSF4-3^T^; 4, *M. tractuosa* DSM 4126^T^; 5, *M. harenae* JK11^T^; 6, *M. sericea* DSM 4125^T^; 7, *M. atlantica* SM 1354^T^; 8, *M. lumbricoides* CGMCC 1.10832^T^; 9, *M. aurantiaca* S37H4^T^.

| **Strains** | **Genome Size (Mbp)** | **Number of Genes** | **Number of CAZyme** | **(%) of CAZyme** | **Number of GHs** | **Ratio of GHs/Mbp** |
| --- | --- | --- | --- | --- | --- | --- |
| **1** | 4.02 | 3471 | 53 | 1.52 | 17 | 4.23 |
| **2** | 3.94 | 3417 | 56 | 1.64 | 21 | 5.33 |
| **3** | 4.49 | 3807 | 59 | 1.55 | 16 | 3.56 |
| **4** | 4.51 | 3800 | 65 | 1.71 | 25 | 5.54 |
| **5** | 4.34 | 3738 | 62 | 1.66 | 22 | 5.07 |
| **6** | 4.74 | 4082 | 72 | 1.76 | 25 | 5.27 |
| **7** | 4.19 | 3630 | 70 | 1.93 | 30 | 7.16 |
| **8** | 5.93 | 4978 | 152 | 3.05 | 75 | 12.65 |
| **9** | 4.2 | 3628 | 74 | 2.04 | 28 | 6.67 |

**Table S5.** Number of polysaccharide-degrading gene families based on the dbCAN meta server, and *in vitro* activities of three isolated strains within the genus *Marivirga*

Strains: 1, BKB1-2^T^; 2, ABR2-2; 3, BDSF4-3^T^

| **Strains** | **Polysaccharides** | ***CAZyme families** | ***in vitro* polysaccharide degradation activities** |
| --- | --- | --- | --- |
| **1** | Agarose | GH50 (0) | - |
|  | Alginate | PL6 (0), PL7 (0) | - |
|  | Cellulose | GH5 (0), GH9 (0) | + |
|  | Chitin | GH3 (3) GH23 (3) | + |
|  | κ-Carrageenan | GH16 (2) | - |
|  | λ-carrageenan | GH110 (0) | - |
|  | ι-carrageenan | GH82 (0) | - |
|  | Laminarin | GH16 (2) | + |
|  | Starch | GH13 (3) GH16 (2) | + |
|  | Xylan | GH3 (3) | - |
| **2** | Agarose | GH50 (0) | - |
|  | Alginate | PL6 (0), PL7 (0) | - |
|  | Cellulose | GH5 (0), GH9 (0) | - |
|  | Chitin | GH3 (5) GH23 (2) | + |
|  | κ-Carrageenan | GH16 (3) | - |
|  | Laminarin | GH16 (3) | + |
|  | λ-carrageenan | GH110 (0) | - |
|  | ι-carrageenan | GH82 (0) | - |
|  | Starch | GH13 (3) GH16 (3) | + |
|  | Xylan | GH3 (5) | - |
| **3** | Agarose | GH50 (0) | - |
|  | Alginate | PL6 (0), PL7 (0) | - |
|  | Cellulose | GH5 (0), GH9 (0) | - |
|  | Chitin | GH3 (2) GH23 (2) | + |
|  | κ-Carrageenan | GH16 (6) | - |
|  | Laminarin | GH16 (2) | + |
|  | λ-carrageenan | GH110 (0) | - |
|  | ι-carrageenan | GH82 (0) | - |
|  | Starch | GH13 (2) GH57 (1) | + |
|  | Xylan | GH3 (2) | + |

*****CAZyme families with the number of genes in parentheses that are expected to participate in degradation of the polysaccharides. GH, glycoside hydrolase; PL, polysaccharide lyase

+, degradation of polysaccharide; -, no degradation of polysaccharide
